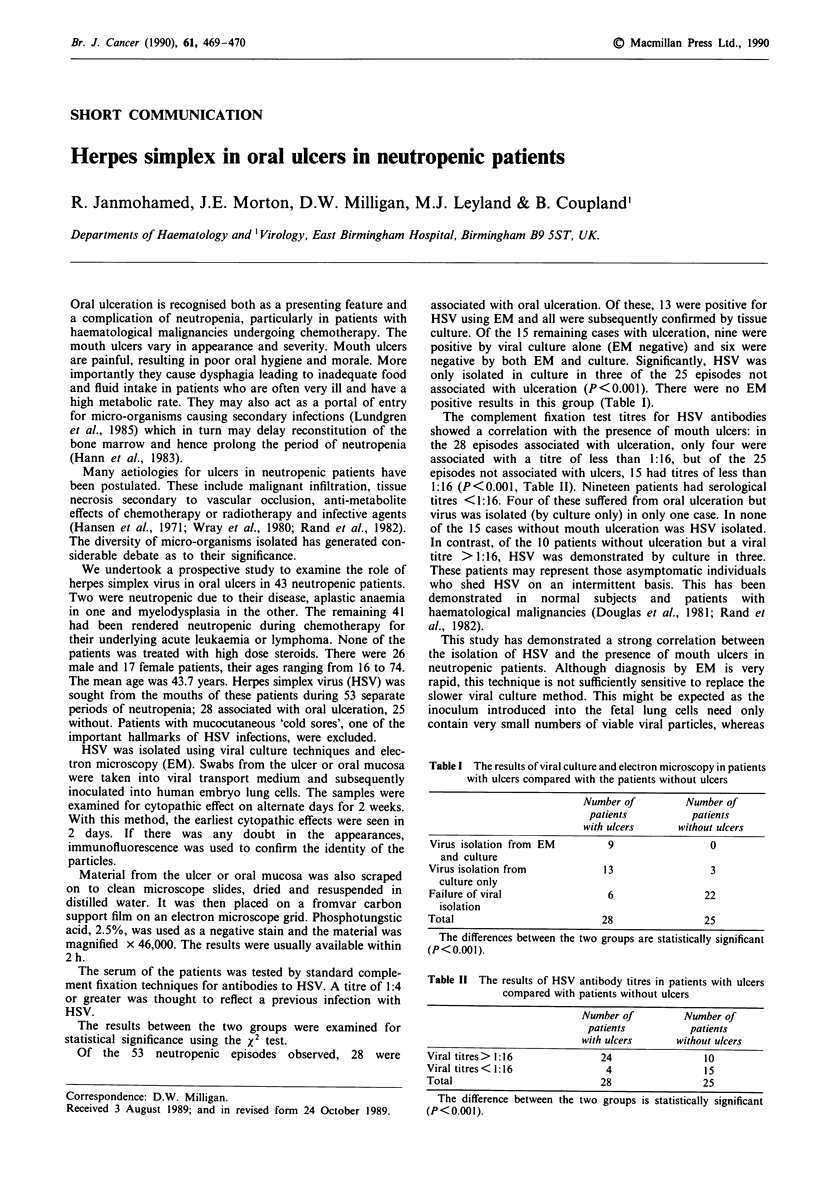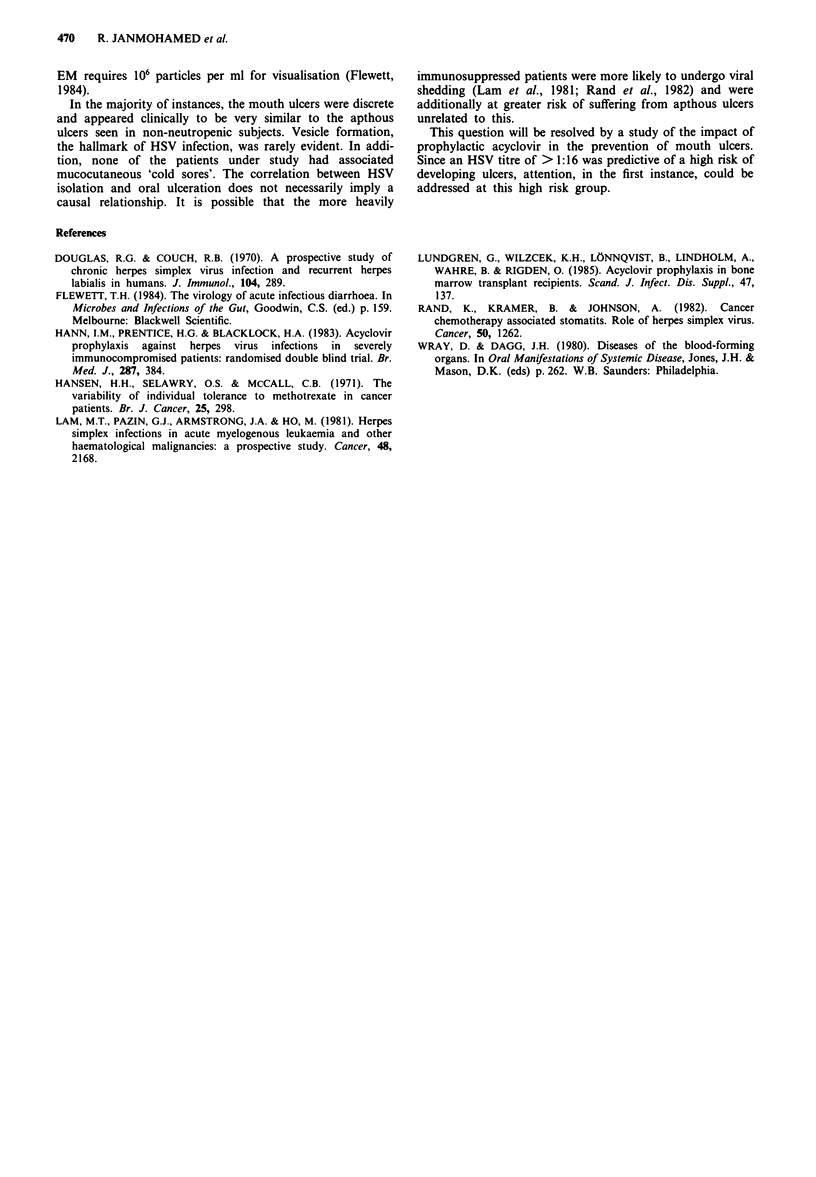# Herpes simplex in oral ulcers in neutropenic patients.

**DOI:** 10.1038/bjc.1990.103

**Published:** 1990-03

**Authors:** R. Janmohamed, J. E. Morton, D. W. Milligan, M. J. Leyland, B. Coupland

**Affiliations:** Department of Haematology, East Birmingham Hospital, UK.


Br. J. Cancer (1990), 61, 469–470 © Macmillan Press Ltd., 1990

SHORT COMMUNICATION

### Herpes simplex in oral ulcers in neutropenic patients

R. Janmohamed, J.E. Morton, D.W. Milligan, M.J. Leyland & B. Coupland[1]

*Departments of Haematology and [1]Virology, East Birmingham Hospital, Birmingham B9 5ST, UK.*

Oral ulceration is recognised both as a presenting feature and a complication of neutropenia, particularly in patients with haematological malignancies undergoing chemotherapy. The mouth ulcers vary in appearance and severity. Mouth ulcers are painful, resulting in poor oral hygiene and morale. More importantly they cause dysphagia leading to inadequate food and fluid intake in patients who are often very ill and have a high metabolic rate. They may also act as a portal of entry for micro-organisms causing secondary infections (Lundgren *et al.*, 1985) which in turn may delay reconstitution of the bone marrow and hence prolong the period of neutropenia (Hann *et al.*, 1983).

Many aetiologies for ulcers in neutropenic patients have been postulated. These include malignant infiltration, tissue necrosis secondary to vascular occlusion, anti-metabolite effects of chemotherapy or radiotherapy and infective agents (Hansen *et al.*, 1971; Wray *et al.*, 1980; Rand *et al.*, 1982). The diversity of micro-organisms isolated has generated considerable debate as to their significance.

We undertook a prospective study to examine the role of herpes simplex virus in oral ulcers in 43 neutropenic patients. Two were neutropenic due to their disease, aplastic anaemia in one and myelodysplasia in the other. The remaining 41 had been rendered neutropenic during chemotherapy for their underlying acute leukaemia or lymphoma. None of the patients was treated with high dose steroids. There were 26 male and 17 female patients, their ages ranging from 16 to 74. The mean age was 43.7 years. Herpes simplex virus (HSV) was sought from the mouths of these patients during 53 separate periods of neutropenia; 28 associated with oral ulceration, 25 without. Patients with mucocutaneous 'cold sores', one of the important hallmarks of HSV infections, were excluded.

HSV was isolated using viral culture techniques and electron microscopy (EM). Swabs from the ulcer or oral mucosa were taken into viral transport medium and subsequently inoculated into human embryo lung cells. The samples were examined for cytopathic effect on alternate days for 2 weeks. With this method, the earliest cytopathic effects were seen in 2 days. If there was any doubt in the appearances, immunofluorescence was used to confirm the identity of the particles.

Material from the ulcer or oral mucosa was also scraped on to clean microscope slides, dried and resuspended in distilled water. It was then placed on a fromvar carbon support film on an electron microscope grid. Phosphotungstic acid, 2.5%, was used as a negative stain and the material was magnified × 46,000. The results were usually available within 2 h.

The serum of the patients was tested by standard complement fixation techniques for antibodies to HSV. A titre of 1:4 or greater was thought to reflect a previous infection with HSV.

The results between the two groups were examined for statistical significance using the $\chi^2$ test.

Of the 53 neutropenic episodes observed, 28 were associated with oral ulceration. Of these, 13 were positive for HSV using EM and all were subsequently confirmed by tissue culture. Of the 15 remaining cases with ulceration, nine were positive by viral culture alone (EM negative) and six were negative by both EM and culture. Significantly, HSV was only isolated in culture in three of the 25 episodes not associated with ulceration ($P<0.001$). There were no EM positive results in this group (Table I).

The complement fixation test titres for HSV antibodies showed a correlation with the presence of mouth ulcers: in the 28 episodes associated with ulceration, only four were associated with a titre of less than 1:16, but of the 25 episodes not associated with ulcers, 15 had titres of less than 1:16 ($P<0.001$, Table II). Nineteen patients had serological titres <1:16. Four of these suffered from oral ulceration but virus was isolated (by culture only) in only one case. In none of the 15 cases without mouth ulceration was HSV isolated. In contrast, of the 10 patients without ulceration but a viral titre >1:16, HSV was demonstrated by culture in three. These patients may represent those asymptomatic individuals who shed HSV on an intermittent basis. This has been demonstrated in normal subjects and patients with haematological malignancies (Douglas *et al.*, 1981; Rand *et al.*, 1982).

This study has demonstrated a strong correlation between the isolation of HSV and the presence of mouth ulcers in neutropenic patients. Although diagnosis by EM is very rapid, this technique is not sufficiently sensitive to replace the slower viral culture method. This might be expected as the inoculum introduced into the fetal lung cells need only contain very small numbers of viable viral particles, whereas

**Table I** The results of viral culture and electron microscopy in patients with ulcers compared with the patients without ulcers

| | *Number of patients with ulcers* | *Number of patients without ulcers* |
|---|---|---|
| Virus isolation from EM and culture | 9 | 0 |
| Virus isolation from culture only | 13 | 3 |
| Failure of viral isolation | 6 | 22 |
| Total | 28 | 25 |

The differences between the two groups are statistically significant ($P<0.001$).

**Table II** The results of HSV antibody titres in patients with ulcers compared with patients without ulcers

| | *Number of patients with ulcers* | *Number of patients without ulcers* |
|---|---|---|
| Viral titres > 1:16 | 24 | 10 |
| Viral titres < 1:16 | 4 | 15 |
| Total | 28 | 25 |

The difference between the two groups is statistically significant ($P<0.001$).

Correspondence: D.W. Milligan.
Received 3 August 1989; and in revised form 24 October 1989.

EM requires $10^6$ particles per ml for visualisation (Flewett, 1984).

In the majority of instances, the mouth ulcers were discrete and appeared clinically to be very similar to the apthous ulcers seen in non-neutropenic subjects. Vesicle formation, the hallmark of HSV infection, was rarely evident. In addition, none of the patients under study had associated mucocutaneous 'cold sores'. The correlation between HSV isolation and oral ulceration does not necessarily imply a causal relationship. It is possible that the more heavily immunosuppressed patients were more likely to undergo viral shedding (Lam et al., 1981; Rand et al., 1982) and were additionally at greater risk of suffering from apthous ulcers unrelated to this.

This question will be resolved by a study of the impact of prophylactic acyclovir in the prevention of mouth ulcers. Since an HSV titre of $>1{:}16$ was predictive of a high risk of developing ulcers, attention, in the first instance, could be addressed at this high risk group.

### References

DOUGLAS, R.G. & COUCH, R.B. (1970). A prospective study of chronic herpes simplex virus infection and recurrent herpes labialis in humans. *J. Immunol.*, **104**, 289.

FLEWETT, T.H. (1984). The virology of acute infectious diarrhoea. In *Microbes and Infections of the Gut*, Goodwin, C.S. (ed.) p. 159. Melbourne: Blackwell Scientific.

HANN, I.M., PRENTICE, H.G. & BLACKLOCK, H.A. (1983). Acyclovir prophylaxis against herpes virus infections in severely immunocompromised patients: randomised double blind trial. *Br. Med. J.*, **287**, 384.

HANSEN, H.H., SELAWRY, O.S. & MCCALL, C.B. (1971). The variability of individual tolerance to methotrexate in cancer patients. *Br. J. Cancer*, **25**, 298.

LAM, M.T., PAZIN, G.J., ARMSTRONG, J.A. & HO, M. (1981). Herpes simplex infections in acute myelogenous leukaemia and other haematological malignancies: a prospective study. *Cancer*, **48**, 2168.

LUNDGREN, G., WILZCEK, K.H., LÖNNQVIST, B., LINDHOLM, A., WAHRE, B. & RIGDEN, O. (1985). Acyclovir prophylaxis in bone marrow transplant recipients. *Scand. J. Infect. Dis. Suppl.*, 47, 137.

RAND, K., KRAMER, B. & JOHNSON, A. (1982). Cancer chemotherapy associated stomatits. Role of herpes simplex virus. *Cancer*, **50**, 1262.

WRAY, D. & DAGG, J.H. (1980). Diseases of the blood-forming organs. In *Oral Manifestations of Systemic Disease*, Jones, J.H. & Mason, D.K. (eds) p. 262. W.B. Saunders: Philadelphia.